# Novel pathological predictive factors for extranodal extension in oral squamous cell carcinoma: a retrospective cohort study based on tumor budding, desmoplastic reaction, tumor-infiltrating lymphocytes, and depth of invasion

**DOI:** 10.1186/s12885-022-09393-8

**Published:** 2022-04-13

**Authors:** Yuri Noda, Mitsuaki Ishida, Yasuhiro Ueno, Takuo Fujisawa, Hiroshi Iwai, Koji Tsuta

**Affiliations:** 1 Department of Pathology and Laboratory Medicine, Kansai Medical University Hospital, 2-3-1 Shin-machi, Hirakata, Osaka 573-1191 Japan; 2 Department of Radiology, Kansai Medical University Hospital, 2-3-1 Shinmachi, Hirakata, Osaka 573-1191 Japan; 3 Department of Otolaryngology, Head and Neck Surgery, Kansai Medical University Hospital, 2-3-1 Shinmachi, Hirakata, Osaka 573-1191 Japan

**Keywords:** Extranodal extension, Oral squamous cell carcinoma, Tumor budding, Desmoplastic reaction, Tumor-infiltrating lymphocytes, Predictor

## Abstract

**Background:**

Extranodal extension (ENE) is a poor prognostic factor for oral squamous cell carcinoma (OSCC). Identifying ENE by clinical and/or radiological examination is difficult, thereby leading to unnecessary neck dissections. Currently, no definitive predictors are available for ENE. Thus, we aimed to determine the histological predictors of ENE by routine histopathological examination using biopsy and surgically resected specimens.

**Methods:**

This retrospective study included 186 surgically resected OSCC and 83 matched biopsy specimens. Clinical features associated with the tumor microenvironment, including desmoplastic reaction (DR), tumor budding (TB), and tumor-infiltrating lymphocytes (TILs), were evaluated using hematoxylin and eosin-stained primary OSCC and neck dissection specimens. These histological features were divided into two groups: DR-immature (DR-I) and DR-mature (DR-M); TB-high (TB-H) and TB-low (TB-L); and TILs-low (TILs-L) and TILs-high (TILs-H). Clinical depth of invasion (cDOI) and pathological DOI (pDOI) were adapted for biopsies and resections, respectively; DOI was evaluated as DOI > 10 mm and DOI ≤ 10 mm. The clinicopathological relationships between these histopathological features and ENE and the independent risk factors for ENE were analyzed. The histological predictors of ENE were evaluated.

**Results:**

The histological status of DR, TILs, and TB present in biopsy and resection specimens showed high accuracy with that of ENE. DR-I, TILs-L, and TB-H were significantly associated with lymph node metastasis, cDOI, and pDOI. Bivariate and multivariate analyses revealed that TB-H and pDOI > 10 mm in resections were independent factors for the presence of ENE (ENE +). The combination of TB-H/pDOI > 10 mm in resection specimens showed high specificity (91%) and accuracy (83%) regarding ENE + . Although there proved to be no independent factors in biopsies, DR-I and TILs-L were significantly associated with ENE + (*p* < 0.001). The combination of DR-I/TILs-L/cDOI > 10 mm in biopsies exhibited high sensitivity and specificity with ENE + (70% and 77%, respectively, *p* < 0.001). These histological predictors could detect even minor ENE (< 2 mm).

**Conclusions:**

The tumor microenvironment status in primary OSCC was significantly associated with that of ENE, and TB-H was an independent risk factor for ENE. The histological status of DR-I/TILs-L/cDOI > 10 mm in biopsy specimens and TB-H/pDOI > 10 mm in resection specimens is a useful predictor of ENE.

**Supplementary Information:**

The online version contains supplementary material available at 10.1186/s12885-022-09393-8.

## Background

Head and neck squamous cell carcinoma (HNSCC), including oral squamous cell carcinoma (OSCC), is the eighth most common cancer worldwide [1]. In Japan, oral and pharynx cancers were diagnosed in over 22,500 people (17.8 per 100.000), and almost 8,000 people (6.3 per 100.000) die annually [2]. Despite advances in cancer diagnosis and treatment, the overall 5-year survival rate for OSCC is still poor, which remains at 63% [3]. Although various prognostic factors of head and neck cancers are known, including cervical lymph node metastasis, distal metastasis, and regional recurrence, the proposed concept of extranodal extension (ENE) is the single-most reliable prognostic clinical variable for mortality, except for human papillomavirus (HPV)-positive cancers (oropharyngeal cancer) [4, 5].

ENE is histopathologically defined as an extension of metastatic carcinoma from within a lymph node, through the fibrous capsule, and into the surrounding connective tissue, regardless of the stromal reaction [6, 7]. The 5-year disease-free survival (DFS) rate of occult lymph node metastasis is quite different in patients with ENE compared with that in patients without ENE (25.8% vs. 71.2%, respectively) [5]. Even patients with OSCC who had minor ENE (ENEmi, an extension of up to 2 mm from the capsule) had worse overall survival (31.0%) and DFS (38.0%) rates than patients without ENE (51.0% and 71.0%, respectively) [7–9]. Considering the impact of ENE on the prognosis, patients with ENE are recommended to undergo neck dissection and to receive chemoradiotherapy after surgery [10, 11]. Accordingly, the 8th edition of the American Joint Committee on Cancer (AJCC) tumor, node, metastasis (TNM) staging system for HNSCC included this factor as an indicator to upgrade the N stage [12].

Although more aggressive treatment is required for patients with ENE, the sensitivity, specificity, and accuracy of clinically defined ENE (cENE) evaluated by physical and/or radiological findings do not achieve a satisfactory level, especially in patients with ENEmi. The accuracy of ENE evaluated by radiological examination ranges widely from 7.0% to < 85.0%, and the detection of ENEmi is almost impossible [9, 13, 14]. Thus, currently, diagnosis requires histological examination of the lymph nodes by neck dissection, although radiological evidence alone may be supportive albeit insufficient for detecting ENE [14]. Therefore, radiologically unclear or ambiguous extracapsular spread may not contribute to an accurate diagnosis, and some patients may undergo treatment or overtreatment for ENE [15, 16]. However, currently, no histopathological predictors that support cENE detection are available.

Several histological predictors for unfavorable prognosis of OSCC, such as tumor-infiltrating lymphocytes (TILs), desmoplastic reaction (DR), tumor budding (TB), and depth of invasion (DOI), have been reported [17–24]. All these components, including immune cells, cancer-associated fibroblasts, and extracellular matrix, are derived from the tumor microenvironment (TME); accordingly, the TME is considered to strongly influence tumor behavior in OSCCs and play an important role in OSCC development [17]. TILs are a selected population of lymphocytes with a highly specific immunological reactivity against tumor cells. DR is characterized by the presence of fibrotic or myxoid stroma induced by tumor invasion and is histopathologically classified into three patterns, namely, mature (no keloid-like collagen or myxoid stroma), intermediate (presence of keloid-like collagen), and immature (presence of myxoid change) [24]. TB is characterized by the presence of small tumor nests composed of less than five cells at the invasion front, which is thought to be related to the metastatic potential of the tumor [21, 22, 25–30]. DOI is defined as the extent of the tumor below the epithelial membrane [6] and can be measured by preoperative radiological assessment using magnetic resonance imaging (MRI) or ultrasonography as clinical DOI (cDOI), which exhibits a high correlation to pathological DOI (pDOI) and pathological T (pT), as evaluated by findings from surgically resected OSCC [6, 31]. It has been widely accepted that low-grade lymphocyte infiltration or immature DR or a high frequency of TB is associated with cancer invasion, metastasis, and poor outcomes in various types of carcinomas, including OSCCs [14–24, 32–34]; moreover, DOI < 10.5 mm is associated with a high incidence of lymph nodes metastasis [35].

However, the significance of these histological predictors in predicting ENE in OSCC has not yet been elucidated. Therefore, in this study, we aimed to identify useful histological factors to support the cENE evaluation and determine whether histological predictors of ENE can be evaluated with biopsies and resections for more appropriate treatment of patients with OSCC.

## Methods

### Patients

This study included patients with OSCC who underwent surgical resection at the Department of Otorhinolaryngology, Head and Neck Surgery, Kansai Medical University Hospital, between January 2011 and December 2020. Cases that met the following criteria were selected: (1) patients with primary OSCC with no clinical history of squamous cell carcinoma (SCC) in other parts of the body and (2) patients who had not received any therapy before surgery included in the present cases. Tumors were histologically diagnosed according to the World Health Organization grading system [36]. Clinical data were collected from the patients’ medical health records, including data on age, sex, tumor site, and cDOI. Histopathological features of surgical resection specimens, including T and N classification, pDOI, number of metastatic nodes, pattern of invasion, and lymphovascular invasion, were re-evaluated by an oral pathologist according to the 8th edition of the AJCC staging manual [6].

Biopsy specimens that met the following criteria were also collected: (1) had an initial histological diagnosis of OSCC, (2) had a sufficient number of SCC cells over a microscopic field of × 200, and (3) had an invasive area accompanying the connective tissue.

This study was conducted in accordance with the principles of the Declaration of Helsinki and was approved by the Institutional Review Board of Kansai Medical University Hospital (approval no. 2020289). Informed consent was obtained from patients using the opt-out methodology, owing to the retrospective design of the study, with no new risk to the participants. Information regarding this study, such as the inclusion criteria and option to opt out, was provided on the hospital’s website.

### Definition and measurement of the diameters of extent of ENE and tumor deposits at the lymph nodes

**Fig. 1:**
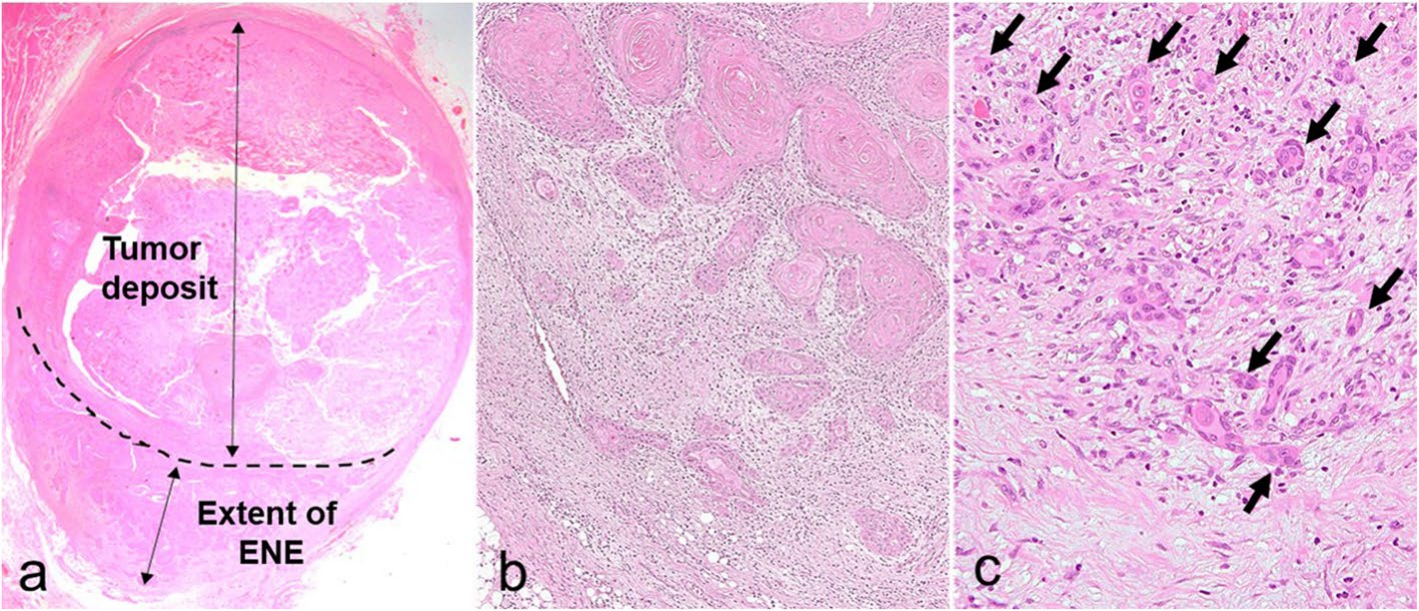
Definition and measurement of ENE and evaluation of DR, TILs, and TB at ENE. **a** Diameters of the extent of ENE and tumor deposits were recorded as the widest length of the dissected lymph nodes of the same patients (H&E staining, × 15). **b**, **c** The ENE region shows immature stroma, low-grade TILs, and nine budding areas (arrows: budding) (H&E staining, b, × 100; c, × 400). DR, desmoplastic reaction; ENE, extranodal extension; H&E, hematoxylin and eosin; TB, tumor budding; TILs, tumor-infiltrating lymphocytes

The ENE was defined as positive if the tumor extension through the lymph node capsule invaded the surrounding connective tissue [6, 7]. The extent of the ENE was measured as the maximum distance between the outer aspect of the intact or reconstructed capsule and the distant point of invasion into the extranodal tissue [7]. The deposit size was measured as the greatest dimension from the foci at one end to the extreme opposite foci [7]. The diameters of the extent of the ENE and tumor deposit were recorded as the widest length of the dissected lymph nodes in the same patients (Fig. 1a) [6, 7].

### Assessment of DR, TB, TILs, and DOI

The DR, TB, and TILs of the primary OSCC were evaluated by staining the biopsy and resection specimen slides with hematoxylin and eosin (H&E). The status of the three pathological features in the ENE foci was also evaluated using the H&E-stained slides of the cervical neck dissection samples. All the H&E-stained slides used in this study were prepared for routine pathological examination. These histological factors were examined for OSCC in the biopsy specimens using a single slide, the resected specimens including the deepest part of the tumor, and the neck dissected specimens including the major ENE part in lymph node metastasis (Figs. 1b, c). The score of the three pathological features was evaluated by two pathologists (NY and IM).

**Fig. 2:**
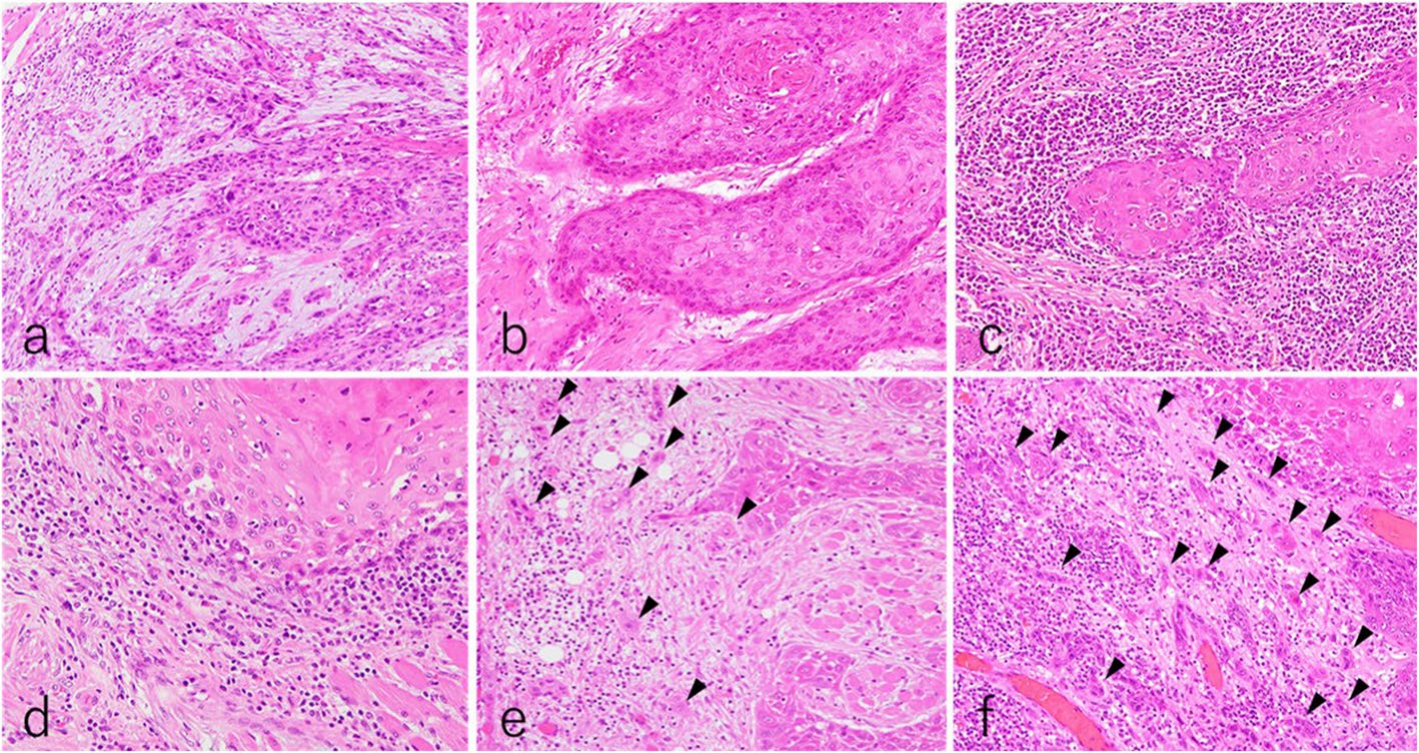
TB, TILs, and TB grading. **a** Immature stroma (DR-I), with a fibrotic stroma showing myxoid changes (H&E staining, × 200). **b**, **c** Mature stroma (DR-M), with no myxoid stroma or keloid-like collagen (H&E staining, × 200). **a**, **b** TILs with low lymphocyte infiltration (TILs-L ≤ 20%) (H&E staining, × 200). **c** TILs with high-to-low lymphocyte infiltration (TILs-H > 20%) (H&E staining, × 200). **d** TB classified as low (TB-L ≤ 10, arrowheads: budding) (H&E staining, × 400). **e**, **f** TB classified as high (TB-H > 10, arrowheads: budding) (H&E staining, × 200). DR-I, immature desmoplastic reaction; DR-M, mature desmoplastic reaction; H&E, hematoxylin and eosin; TB, tumor budding; TILs, tumor-infiltrating lymphocytes

The DR was histologically classified as immature (DR-I) or mature (DR-M) [24]. DR-I refers to a fibrotic stroma with myxoid changes as observed across a microscopic field under a × 40 objective (Fig. 2a), and DR-M refers to the absence of a myxoid stroma or presence of keloid-like collagen lacking mature stroma (Fig. 2b) [24]. The stromal TILs were evaluated as the average number of lymphocytes composed of stromata as observed under × 20 to × 40 objectives; they were categorized as low (TILs-L ≤ 20%) or high (20% < TILs-H) (Figs. 2a–c) [19]. TB was assessed and classified as low (TB-L ≤ 10) or high (10 > TB-H) as observed under a × 20 objective (Figs. 2d–f) [25]. The DOI of primary OSCC was defined as the perpendicular distance between the extent of deep tumor invasion to the basement membrane of the adjacent mucosa [6]. The DOI was subdivided into the following groups: DOI ≤ 5 mm, DOI > 5 mm, DOI ≤ 10 mm, and DOI > 10 mm [6]. cDOI was measured by MRI findings and pDOI histologically**,** and they were then associated with the clinicopathological features and ENE risk factors of the biopsy and resection specimens, respectively. pT1 and pT2 OSCC cases were classified as early OSCC cases and pT3 and pT4 as progressive OSCC cases.

### Statistical analyses

Patient characteristics were compared between the two groups in terms of DR-I and DR-M, TB-H and TB-L, TILs-H and TILs-L, cDOI for biopsies (≤ 10 mm and > 10 mm), and pDOI for resections (≤ 10 mm and > 10 mm), using the chi-square or Fisher’s exact test for categorical data. Bivariate logistic regression analysis was used to assess the relationship between the predictor variables and presence of ENE. A multivariate logistic regression model was constructed using the forward selection method. The diagnostic value of the risk factors was assessed by calculating sensitivity, specificity, positive predictive value (PPV), negative predictive value (NPV), and accuracy. The Mann − Whitney *U* test was used to analyze the differences in the diameters of the extent of ENE and tumor deposit between the combination risk factors. The analysis was performed using SPSS (version 20.0; IBM Corp., Armonk, NY, USA). Statistical significance was set at *p* ≤ 0.05.

## Results

### Patient characteristics of the surgical specimens of OSCC

A total of 674 patients were included in this study; 488 patients were excluded, and 186 who met the criteria were subjected to further analyses. The clinicopathological characteristics of 186 patients with OSCC, including 92 with early and 94 with progressive OSCC, are shown in Table 1 (Additional File 1). Among the 186 patients with OSCC, 123 (66%) had pDOI > 5 mm, and 85 (46%) had pDOI > 10 mm. Fifty-eight (31%) had lymph node metastasis, and 16 and 42 patients were classified as having pN1 and pN2–3, respectively. ENE was detected in 27 (15%) of the 186 patients with OSCC. Overall, 93% (25 of 27) of patients with ENE were in the progressive stage. No high-risk HPV was detected in any patient (Additional Files 2 and 3).

**Table 1:** Patients’ demographics

		All patients (*n* = 186)		Early OSCC (*n* = 92)		Progressive OSCC (*n* = 94)	
Characteristics		No. of patients	%	No. of patients	%	No. of patients	%
Sex	Female	71	(38.1)	36	(39.1)	35	(37.2)
	Male	115	(61.8)	56	(60.9)	59	(62.8)
Age, years	Mean age	68.9 (range, 31 − 91)		69 (range, 31 − 91)		68.6 (range, 38 − 87)	
	≥ 64	57	(30.6)	27	(29.3)	30	(31.9)
	< 64	129	(69.4)	65	(70.7)	64	(68.1)
High-risk HPV exposure	No	186	(100)	92	(100)	94	(100)
Anatomical site	Palate	4	(2.1)	4	(4.3)	0	(0)
	Oral floor	3	(1.6)	2	(2.2)	1	(1.1)
	Gingiva	29	(15.6)	4	(4.3)	25	(26.6)
	Tongue	129	(69.3)	67	(72.8)	62	(66.0)
	Buccal mucosa	21	(11.3)	15	(16.3)	6	(0)
DOI	≤ 5 mm	63	(33.9)	58	(63.0)	5	(5.3)
	> 5 mm	123	(66.1)	34	(40.0)	89	(94.7)
DOI	≤ 10 mm	102	(54.9)	92	(100)	10	(10.6)
	> 10 mm	84	(45.2)	0	(0)	84	(9.4)
LyV	Negative	73	(39.2)	54	(58.7)	19	(20.2)
	Positive	113	(60.8)	38	(41.3)	75	(79.8)
ENE	Negative	159	(85.5)	90	(97.8)	69	(73.4)
	Positive	27	(14.5)	2	(2.2)	25	(26.6)
pT	1, 2	92	(49.5)	92	(100)	0	(0)
	3, 4	94	(50.5)	0	(0)	94	(100)
pN	0	128	(68.8)	83	(90.2)	45	(47.9)
	1	16	(8.6)	4	(4.3)	12	(64.5)
	2, 3	42	(22.6)	5	(5.4)	37	(19.9)

*OSCC* Oral squamous cell carcinoma, *HPV* Human papilloma virus, *DOI* Depth of invasion, *LyV* Lymphovascular invasion, *ENE* Extranodal extension, *pT* pathological T, *pN* pathological N. DOI included both cDOI and pDOI because the cohorts of clinical DOI and pathological DOI were matched 100% (concordance rate = 100%); cutoff points were DOI ≤ 5 mm/DOI > 5 mm and DOI ≤ 10 mm/DOI > 10 mm

### Association of the clinicopathological features with DR, TB, and TILs in the resected specimens

Table 2 shows the association of the clinicopathological features with DR, TB, and TILs in the resected specimens. DR-I, TB-H, and TILs-L were significantly associated with pDOI > 10 mm (*p* < 0.001, 0.01, and < 0.001, respectively) and pT stage (1, 2 versus 3, 4) (*p* < 0.001, 0.01, and < 0.001, respectively). These three factors were also significantly associated with the presence of lymphovascular invasion and lymph node metastasis (all *p* < 0.05). Moreover, infiltrative growth was significantly associated with DR-I, TB-H, and TILs-L (*p* < 0.001*,* 0.01, and < 0.01, respectively). However, tumor location was not significantly associated with DR, TB, or TILs.

**Table 2:** Association of clinicopathological features with DR, TB, and TILs using surgically resected specimens

	Desmoplastic reaction					Tumor budding					Tumor-infiltrating lymphocytes				
	Mature	(%)	Immature	(%)	*p-value*	Low	(%)	High	(%)	*p-value*	High	(%)	Low	%	*p-value*
Sex					0.81					0.19					0.06
Female	29	(41)	42	(59)		53	(75)	18	(25)		44	(62)	27	(38)	
Male	49	(43)	66	(57)		95	(83)	20	(17)		55	(48)	60	(52)	
Age, years					0.17					** < 0.001**					0.59
< 65	28	(49)	29	(50)		41	(72)	16	(28)		32	(56)	25	(44)	
≥ 65	50	(39)	79	(61)		107	(83)	22	(17)		67	(52)	62	(48)	
Location					0.63					0.6					0.36
Palate	3	(75)	1	(21)		4	(100)	0	(0)		3	(75)	1	(25)	
Oral floor	1	(33)	2	(60)		3	(100)	0	(0)		2	(67)	1	(33)	
Gingiva	12	(41)	17	(58)		22	(76)	7	(24)		11	(38)	18	(62)	
Tongue	55	(43)	74	(57)		101	(78)	28	(22)		70	(54)	59	(46)	
Buccal mucosa	7	(33)	14	(66)		18	(86)	3	(14)		13	(62)	8	(38)	
pDOI					** < 0.001**					0.06					** < 0.001**
≥ 5 mm	46	(73)	17	(27)		55	(87)	8	(13)		49	(78)	14	(22)	
< 5 mm	32	(26)	91	(74)		93	(76)	30	(24)		50	(41)	73	(59)	
pDOI					** < 0.001**					**0.01**					** < 0.001**
≥ 10 mm	57	(56)	45	(44)		88	(86)	14	(14)		72	(71)	30	(29)	
< 10 mm	21	(25)	63	(75)		60	(71)	24	(29)		27	(32)	57	(68)	
Metastasis					** < 0.001**					**0.02**					** < 0.001**
Absent	68	(53)	60	(47)		108	(84)	20	(16)		79	(62)	49	(38)	
Present	10	(17)	48	(83)		40	(69)	18	(31)		20	(34)	38	(66)	
Number of LNs					** < 0.001**					**0.03**					** < 0.001**
* n* = 0	68	(53)	60	(47)		108	(84)	20	(16)		79	(62)	49	(38)	
* n* = 1	5	(23)	16	(76)		16	(76)	5	(24)		10	(48)	11	(52)	
* n* > 2	5	(13)	32	(86)		24	(65)	13	(35)		10	(27)	27	(73)	
Pattern of invasion					** < 0.001**					**0.01**					** < 0.01**
1, 2	30	(70)	13	(30)		40	(93)	3	(7)		31	(72)	12	(28)	
3, 4	48	(34)	95	(66)		108	(76)	35	(24)		68	(48)	75	(52)	
LyV					** < 0.001**					**0.03**					** < 0.001**
Absent	44	(60)	29	(39)		64	(88)	9	(12)		50	(68)	23	(32)	
Present	34	(30)	79	(70)		84	(74)	29	(26)		49	(43)	64	(57)	
pT					** < 0.001**					**0.01**					** < 0.001**
1, 2	54	(59)	38	(41)		80	(87)	12	(13)		67	(73)	25	(27)	
3. 4	24	(26)	70	(74)		68	(72)	26	(28)		32	(34)	62	(66)	
pN					** < 0.001**					**0.02**					** < 0.01**
0	68	(53)	60	(47)		108	(84)	20	(16)		79	(42)	49	(41)	
1,	3	(19)	13	(81)		13	(81)	3	(19)		6	(37)	10	(63)	
2, 3	7	(17)	35	(83)		27	(64)	15	(36)		14	(33)	28	(67)	

*OSCC* Oral squamous cell carcinoma, *DR* Desmoplastic reaction, *TB* Tumor budding, *TILs* Tumor-infiltrating lymphocytes, *pDOI* pathological depth of invasion, *LN* lymph nodes, *LyV* lymphovascular invasion, *pT* pathological T, *pN* pathological N. **Boldface** indicates statistically significant values

### Association among DR, TB, and TILs in the resected specimens and ENE

The results of the association among DR, TB, and TILs in the resected specimens and ENE sites are shown in Table 3. These three pathological features of the resection specimens were significantly associated with those of the ENE sites (all *p* < 0.05).

**Table 3:** Relationships among DR, TB, and TILs in ENE and resection (*n* = 27)

		In matched ENE (*n* = 27)			
		DR-M	DR-I	CR	*p-value*
**In matched resection (*n*** **= 27)**	**DR-M**	3	2	89%	** < *0.01***
	**DR-I**	1	21		
		TB-L	TB-H	CR	*p-value*
	**TB-L**	13	2	74%	***0.01***
	**TB-H**	5	7		
		TILs-H	TILs-L	CR	*p-value*
	**TILs-H**	13	4	78%	** < *0.01***
	**TILs-L**	2	8		

*DR* Desmoplastic reaction, *TB* Tumor budding, *TILs* Tumor-infiltrating lymphocytes, *ENE* Extranodal extension, *CR* Concordance rate, *DR-I* Immature desmoplastic reaction, *DR-M* Mature desmoplastic reaction, *TB-H* High tumor budding, *TB-L* Low tumor budding, *TILs-L* Low-grade tumor-infiltrating lymphocytes, *TILs-H* High-grade tumor-infiltrating lymphocyte

Based on the clinicopathological analysis of DR, TB, and TILs in the resected specimens and association analysis with ENE, it was indicated that evaluating DR, TB, and TILs in the primary sites, as well as from ENE, could predict tumor behavior.

### Patient characteristics of the OSCC in matched biopsies

We subsequently examined whether biopsy-derived DR, TB, and TILs would be useful histopathological factors to predict tumor behavior. A total of 131 biopsies were collected from the matched patients, and 83 biopsies (51%, 83 of 186), including 36 from patients with early and 47 from those with progressive OSCC, met the criteria and were then subjected to further analyses (Additional File 4). Of the 83 biopsies, 27 (33%) had lymph nodes metastasis, and 10 patients with ENE were available (12%, 10/83), all of whom were in the progressive stage. cDOI was unavailable in three of 186 OSCC cases (SCC 103, SCC 152, and SCC 163); two had artifacts, and one was evaluated by computed tomography. Among the 183 cDOI evaluated cases, the concordance rate of DOI ≤ 5 mm and DOI > 5 mm and of DOI ≤ 10 mm and DOI > 10 mm between cDOI and pDOI was 100% (Supplementary Table 1 in Additional File 1). However, the three unavailable cases were not included in the analysis of biopsy specimens; accordingly, cDOI was applicable in all 83 biopsies, 54 (66%) of which had cDOI > 5 mm and 41 (48%) had cDOI ≤ 10 mm.

### Association of the clinicopathological features with DR, TB, and TILs in biopsy specimens

Table 4 summarizes the association of clinicopathological features with DR, TB, and TILs in biopsy specimens. DR-I and TILs-L were significantly associated with cDOI > 5 mm, cDOI > 10 mm, and pT stage (all *p* < 0.01) similar to that in resected specimens, although TB was not significantly associated. DR-I and TILs-L were also significantly associated with the presence and number of lymph node metastases (all *p* < 0.05). TB-H was significantly associated with the number of metastatic lymph nodes (*p* = 0.02). DR, TB, and TILs were not significantly associated with tumor location.

**Table 4:** Association of clinicopathological features with DR, TB, and TILs in biopsy specimens

	Desmoplastic reaction					Budding					Tumor-infiltrating lymphocytes				
	Mature	(%)	Immature	(%)	*p-value*	Low	(%)	High	(%)	*p-value*	High	(%)	Low	%	*p-value*
Sex					0.44					0.62					0.5
Female	13	(39)	20	(61)		26	(79)	7	(21)		19	(58)	14	(42)	
Male	24	(48)	26	(52)		37	(74)	13	(26)		25	(50)	25	(50)	
Age, years					0.53					0.51					0.68
< 63	12	(40)	18	60)		24	(80)	6	(20)		15	(50)	15	(50)	
≥ 64	25	(47)	28	(53)		39	(74)	14	(26)		29	(55)	24	(45)	
Location					0.7					0.6					0.73
Palate	1	(100)	0	(0)		1	(100)	0	(0)		1	(100)	0	(0)	
Oral floor	0	(0)	0	(0)		0	(0)	0	(0)		0	(0)	0	(0)	
Gingiva	6	(43)	8	(57)		12	(86)	2	(14)		7	(50)	7	(50)	
Tongue	26	(43)	34	(57)		45	(75)	15	(25)		31	(52)	29	(48)	
Buccal mucosa	4	(50)	4	(50)		5	(62)	3	(38)		5	(63)	3	(38)	
cDOI					** < 0.001**					0.28					** < 0.01**
≤ 5 mm	25	(86)	4	(14)		24	(83)	5	(17)		22	(76)	7	(24)	
> 5 mm	12	(22)	42	(78)		39	(72)	15	(328)		22	(41)	32	(59)	
cDOI					** < 0.001**					**0.1**					** < 0.001**
≤ 10 mm	29	(69)	13	(31)		35	(83)	7	(17)		31	(74)	11	(26)	
> 10 mm	8	(20)	33	(80)		28	(68)	13	(32)		13	(32)	28	(68)	
Metastasis					** < 0.01**					**0.41**					** < 0.01**
Absent	31	(55)	25	(45)		44	(79)	12	(21)		36	(64)	20	(36)	
Present	6	(22)	21	(78)		19	(70)	8	(30)		8	(30)	19	(70)	
Number of LNs					**0.02**					**0.049**					**0.01**
* n* = 0	31	(55)	25	(45)		44	(79)	12	(21)		36	(64)	20	(36)	
* n* = 1	2	(17)	10	(83)		11	(92)	1	(8)		3	(25)	9	(75)	
* n*≧2	4	(27)	11	(73)		8	(53)	7	(47)		5	(33)	10	(67)	
pT					** < 0.001**					0.06					** < 0.001**
1, 2	27	(75)	9	(25)		31	(86)	5	(14)		27	(75)	9	(25)	
3, 4	10	(21)	37	(79)		32	(68)	15	(32)		17	(36)	30	(64)	
pN					**0.02**					**0.2**					**0.01**
0	31	(55)	25	(45)		44	(79)	12	(21)		36	(64)	20	(36)	
1	2	(22)	7	(78)		8	(89)	1	(11)		2	(22)	7	(78)	
2, 3	4	(22)	14	(78)		11	(61)	7	(39)		6	(33)	12	(67)	

*DR* Desmoplastic reaction, *TB* Tumor budding, *TILs* Tumor-infiltrating lymphocytes, *cDOI* clinical depth of invasion, *LN* lymph nodes, *pT* pathological T, *pN* pathological N

### Association among DR, TB, and TILs in biopsies and ENE sites

Similar to resections, the three pathological features of biopsies were significantly associated with those of the ENE site (all *p* < 0.05, Table 5). In addition, these three features in biopsies were also significantly associated with matched resections (all *p* ≤ 0.001, Table 6).

**Table 5:** Relationships among DR, TB, and TILs in ENE and biopsy (*n* = 10)

		In matched ENE (*n* = 10)			
		DR-M	DR-I	CR	*p-value*
**In matched biopsy (*n*** **= 10)**	**DR-M**	0	0	100%	―
	**DR-I**	0	10		
		TB-L	TB-H	CR	*p-value*
	**TB-L**	5	1	90%	***0.02***
	**TB-H**	0	4		
		TILs-H	TILs-L	CR	*p-value*
	**TILs-H**	1	1	90%	***0.03***
	**TILs-L**	0	8		

*DR* Desmoplastic reaction, *TB* Tumor budding, *TILs* Tumor-infiltrating lymphocytes, *ENE* Extranodal extension, *CR* Concordance rate, *DR-I* Immature desmoplastic reaction, *DR-M* Mature desmoplastic reaction, *TB-H* High tumor budding, *TB-L* Low tumor budding, *TILs-L* Low-grade tumor-infiltrating lymphocytes, *TILs-H* High-grade tumor-infiltrating lymphocytes

**Table 6:** Relationships among DR, TB, and TILs in biopsy and resection (*n* = 83)

		In matched biopsy (*n* = 83)			
		DR-M	DR-I	CR	*p-value*
**In matched resection (*n* = 83)**	**DR-M**	30	7	83%	** < *0.001***
	**DR-I**	7	39		
		TB-L	TB-H	CR	*p-value*
	**TB-L**	55	11	77%	***0.001***
	**TB-H**	8	9		
		TILs-H	TILs-L	CR	*p-value*
	**TILs-H**	36	8	82%	** < *0.001***
	**TILs-L**	8	31		

*DR* Desmoplastic reaction, *TB* Tumor budding, *TILs* Tumor-infiltrating lymphocytes, *ENE* Extranodal extension, *CR* Concordance rate, *DR-I* Immature desmoplastic reaction, *DR-M* Mature desmoplastic reaction, *TB-H* High tumor budding, *TB-L* Low tumor budding, *TILs-L* Low-grade tumor-infiltrating lymphocytes, *TILs-H* High-grade tumor-infiltrating lymphocytes

Based on the results, similar to that in the resected specimens, histopathological evaluation of DR, TB, and TILs in a biopsy was proven useful for predicting the clinicopathological features and tumor behavior, including metastatic potential and invasiveness, at ENE in metastatic lymph nodes.

### Predictive factors for the presence of ENE and the predictive value of single or combined risk factors for resected specimens

Evaluating the histological factors of DR, TB, and TILs in both biopsies and resections could predict tumor behavior, including lymph node metastasis activity and tumor behavior in ENE; therefore, we performed bivariate and multivariate analyses to evaluate the predictive factors for detecting ENE in resected specimens. To evaluate the predictive factors for ENE, we performed the analyses in all patients, those with progressive OSCC and those with lymph node metastasis. The bivariate analysis showed that the independent variables for predicting ENE were TB-H (odds ratio [OR] = 3.30, 95% confidence interval [CI] = 1.15 − 9.60; *p* = 0.03) and pDOI > 10 mm (OR = 6.90, 95% CI = 1.49 − 31.9; *p* = 0.01) in all patients; TB-H (OR = 1.04, 95% CI = 1.37 − 13.3; *p* = 0.01) in those with progressive OSCC; and TB-H (OR = 5.70, 95% CI = 1.39 − 23.4; *p* = 0.02) in those with lymph node metastasis (Table 7). However, the multivariate logistic regression revealed that TB-H (OR = 3.14, 95% CI = 1.25 − 7.91; *p* < 0.001) and pDOI > 10 mm (OR = 8.05, 95% CI = 2.62 − 24.7; *p* < 0.001) were independent factors for ENE (Table 7). In the biopsies, bivariate logistic regression analyses revealed no significant relationships between the risk factors and presence of ENE (Additional File 5).

**Table 7:** Bivariate and multivariate logistic regression analyses of ENE using surgically resected specimens

	Bivariate			Multivariate		
	Odds ratio	95% CI	*p*	Odds ratio	95% CI	*p*
All (*n* = 186)						
DR-I	1.71	0.52 − 5.63	0.38			
TB-H	3.30	1.15 − 9.60	**0.03**	3.14	1.25 − 7.91	** < 0.001**
TILs-L	1.74	0.59 − 5.12	0.31			
pDOI > 5 mm	1.47	0.18 − 11.7	0.71			
pDOI > 10 mm	6.90	1.49 − 31.9	**0.01**	8.05	2.62 − 24.7	** < 0.001**
Progressive (*n* = 94)						
DR-I	1.04	0.29 − 3.68	0.94			
TB-H	1.04	1.37 − 13.3	**0.01**			
TILs-L	1.59	0.50 − 5.02	0.42			
pDOI > 5 mm	1.06	0.04 − 27.4	0.97			
pDOI > 10 mm	1.40	0.13 − 14.4	0.67			
Metastasis (*n* = 56)						
DR-I	0.58	0.13–2.66	0.48			
TB-H	5.70	1.39 − 23.4	**0.02**			
TILs-L	2.37	0.64 − 9.94	0.20			
pDOI > 5 mm	0.31	0.02 − 4.40	0.38			
pDOI > 10 mm	2.99	0.50 − 17.9	0.23			

*CI* Confidence interval, *DR-I* Immature desmoplastic reaction, *TB-H* High tumor budding, *TILs-L* Low-grade tumor-infiltrating lymphocytes, *pDOI* pathological depth of invasion, *ENE* Extranodal extension

### Association between pathological high-risk factors and the presence of ENE

The sensitivity, specificity, PPV, NPV, and accuracy for single or a combination of independent factors for pathological features in detecting ENE were analyzed (Table 8). In the resected specimens, TB-H showed high specificity and low sensitivity for the presence of ENE (sensitivity, 44%; specificity, 84%; accuracy, 78%). In contrast, pDOI > 10 mm exhibited high sensitivity and low specificity (sensitivity, 85%; specificity, 62%; accuracy, 65%). The combination of TB-H and pDOI > 10 mm (TB-H/pDOI > 10 mm) showed the highest specificity and accuracy (sensitivity, 37%; specificity, 91%; accuracy, 83%).

**Table 8:** Prediction of ENE by single and combination risk factors in biopsy and surgically resected specimens

Risk factors	Sensitivity (%)	Specificity (%)	PPV (%)	NPV (%)	Accuracy (%)	*p*	Cohort proportion (n; yes vs. no)
Surgically resected specimens							
TB-H	44	84	33	90	78	** < 0.01**	(38:148)
pDOI > 10 mm	85	62	27	96	65	** < 0.01**	(84:102)
TB-H/pDOI > 10 mm	37	91	42	88	83	** < 0.01**	(24:162)
Biopsy specimens							
DR-I	100	51	22	100	57	** < 0.01**	(46:37)
TILs-L	80	58	21	95	60	**0.03**	(39:44)
TB-H	-	-	-	-	-	0.16	(20:63)
cDOI > 10 mm	90	56	22	98	60	** < 0.01**	(41:42)
DR-I/TILs-L	80	31	26	96	70	** < 0.01**	(31:52)
DR-I/TILs-L/cDOI > 10 mm	70	77	29	95	76	** < 0.01**	(24:59)

*PPV* Positive predictive value, *NPV* Negative predictive value, *CR* Concordance rate, *TB-H* High tumor budding, *pDOI* pathological depth of invasion, *DR-I* Immature desmoplastic reaction, *TILs-L* Low-grade tumor-infiltrating lymphocytes, *cDOI* clinical depth of invasion, *ENE* Extranodal extension. **Boldface** indicates statistically significant values

In the biopsy specimens, although no independent histological factors were noted for predicting ENE, significant associations between ENE and the status of DR-I, TILs-L, and radiologically evaluated cDOI >10 mm were detected (all *p*<0.05). The histological features of DR-I, TILs-L, and cDOI >10 mm exhibited high sensitivity but low specificity and low accuracy (all sensitivity, >80%; all specificity, <60%; all accuracy, <60%). The combination of DR-I and TILs-L (DR-I/TILs-L) also showed high sensitivity but low specificity and accuracy (80%, 31%, and 70%, respectively). The combination of DR-I, TILs-L, and cDOI >10 mm (DR-I/TILs-L/cDOI >10 mm) demonstrated >70% sensitivity, 77% specificity, and 76% accuracy. Moreover, TB-H/pDOI>10 mm, DR-I/TILs-L, and DR-I/TILs-L/cDOI>10 mm could identify all ENEmi (100%, 5/5) (Additional File 6).

## Discussion

In this study, we clearly demonstrated that the combination of TB-H/pDOI > 10 mm in resections showed the highest specificity and accuracy for detecting ENE and that the combination of DR-I/TILs-L/cDOI > 10 mm in biopsies showed high sensitivity, specificity, and accuracy for detecting ENE, including ENEmi. These results were derived from DR, TB, and TILs in biopsy specimens, resected specimens, and ENE site, all of which were significantly associated. Moreover, DR-I, TB-H, and TILs-L in resected specimens and DR-I and TILs-L in biopsy specimens were significantly associated with DOI > 10 mm, pT stage, and lymph node metastasis, and TB-H and pDOI > 10 mm in resected specimens were identified as independent factors for the presence of ENE in the multivariate analysis. We confirmed that evaluating the DR, TB, TILs, and DOI in the primary site and a combination of these factors in both resection and biopsies is useful for predicting the tumor behavior in metastatic lymph nodes and presence of ENE in patients with OSCC.

ENE occurs in 37.5 − 56.4% of lymph node metastases in OSCC [37, 38]. Assessing ENE is vital during both pre- and postoperative examinations to decide whether wide-neck resection or chemoradiotherapy should be performed [39]. However, several ENEs are overlooked during clinical examinations due to limited radiological accuracy and the absence of a critical predictor of ENE. Recent studies have shown that *TP53* mutation and SERPINE1 expression in fibroblasts increase the risk of ENE [40, 41]. However, their abilities as predictors of ENE are limited, and the evaluation of these factors is difficult in routine work [40, 41]; thus, a versatile and more simple method for supporting the cENE evaluation and predicting the presence of ENE using biopsy or surgically resected specimens of OSCC is required.

To the best of our knowledge, this study is the first to demonstrate that TB-H, DR-I, and TILs-L and their combination in resections or biopsies are useful predictors for the presence of ENE, including ENEmi. DR, TB, and TILs at the ENE site were significantly correlated with those at the primary sites. This suggests that the TME in the primary sites is similar to that in the ENE sites; the TME affects the tumor behavior in OSCC and indicates the presence of ENE. In other words, histological evaluation of DR, TIL, and BD in the biopsy could predict the tumor behaviors in the resections and ENE. The TME in the primary site might influence the microenvironment in ENE [17, 41]. During carcinogenesis, the synthesis and remodeling of TME components may induce cancer immuno-response and epithelial–mesenchymal transition, which promotes the miniaturization of tumor nests and deposition of reactive stroma [17, 18]. Thus, the TME in the primary sites of OSCC might be related to the presence of lymph node metastasis and ENE [17, 32]. Therefore, additional large-sample studies are needed to establish a nomogram (parameters including TB, DR, TILs, and cDOI in biopsies) to predict the presence of ENE in patients with OSCC, which can aid in providing appropriate treatment, including wide-neck lymph node dissection and/or chemoradiation therapy.

To the best of our knowledge, no reports have yet addressed the association between TB and ENE. This study clearly showed that TB-H was significantly associated with lymph node metastasis and was a powerful independent factor for ENE, showing high specificity in resections but not in biopsies. It is presumed that biopsies do not contain adequate invasion front regions, leading to the low prediction accuracy of ENE. TB is defined as a single cancer cell or a small cluster comprising less than five cancer cells at the tumor invasion front [21–23]. Thus far, several studies have demonstrated that TB is a valuable prognostic marker, especially in the colon, nasopharynx, esophagus, lung, and breast [21, 22, 25–30, 32, 33]. A meta-analysis revealed that high-grade TB is significantly associated with lymph node metastasis in OSCC [21]. These are consistent with our results.

In contrast to TB, DOI in both biopsies and resections showed high sensitivity and low specificity for the presence of ENE, and this study is the first to report that pDOI > 10 mm is an independent risk factor for ENE. DOI is defined as the extent of the tumor below the epithelial membrane, and it can be measured by preoperative radiological assessment using MRI or ultrasonography [6]. High tumor DOI is associated with a high frequency of lymph node metastasis, and especially DOI > 10.5 mm is associated with a high incidence of ENE [35]. Moreover, the high correlation between cDOI and pDOI has been well described [31] and is consistent with the current data; thus, the AJCC 8th edition includes it as a parameter for T staging (≤ 5 mm and > 5 mm; ≤ 10 mm and > 10 mm) [6, 14, 42, 43]. Most ENEs were considered to have occurred in the advanced stage (93%, 25/27) with DOI > 10 mm, and 79% of cases with lymph node metastasis (46 of 58) had a DOI > 10 mm; thus, DOI > 10 mm could increase the sensitivity and/or specificity of factors, such as DR-I/TILs-L and TB-H.

Our study demonstrated the association between DR-I and higher pT and the presence of lymph node metastasis, including ENE. Previous studies have revealed that DR-I is associated with higher pT, presence of lymph node metastasis, and poor outcome in OSCC [17, 21, 23]. This might be due to the transformation of fibroblasts to cancer-associated fibroblasts (CAFs) during carcinogenesis [24]. CAFs promote tumor invasiveness and remodel the stroma from a mature to an immature state by the deposition of glycosaminoglycans through growth differentiation factor (GDF10) secretion [44, 45]. This represents the histopathological features of DR-I [24, 44]. Therefore, the evaluation of DR in both biopsies and resections is a useful predictive factor for tumor behavior and lymph node metastasis in patients with OSCC.

Our results showed that TILs-L was significantly associated with DOI > 5 mm, DOI > 10 mm, lymph node metastasis, and the number of metastatic nodes in resections and biopsies. TILs are a selected population of T-cells with a higher specific immunological reactivity against tumor cells [17, 18]. In OSCC, TILs also include B-cells, and B-cell density is a good prognostic predictor in male and younger patients with OSCC who consume tobacco or alcohol [46]. In addition, TIL is a modulator of cancer invasion and metastasis [17, 18], and low-grade lymphocyte infiltration is associated with worse outcomes [19, 47]; these reports are consistent with the current results.

Currently, some studies examine DR and TIL considering the distribution of tumors nest and stroma; DR and TILs are assessed as the concepts of “tumor-stroma ratio” and “Immune phenotype,” respectively [47, 48]. Stroma-rich OSCC with low infiltration of lymphocytes has an aggressive behavior and is associated with unfavorable prognosis [49], which was consistent with our result, in which DR-I and TILs-L were significantly associated with powerful prognostic factor ENE + .”

In this study, a single pathological risk factor showed high sensitivity but low specificity and vice versa. In biopsies, the combination of DR-I/TILs-L/cDOI > 10 mm successfully had higher sensitivity, specificity, and accuracy (70%, 77%, and 76%, respectively), whereas TB-H/pDOI > 10 mm exhibited higher specificity and accuracy (91% and 83%, respectively) than a single factor in resections.

These predictive factors could be simply evaluated using routine H&E staining and included in pathology reports. Few studies have examined the pathological predictive factors for ENE [40, 41]. Gleber-Netto et al. noted an increasing trend toward the frequency of ENE among patients with *TP53*-mutant OSCC (65.5%) than among those with WT *TP53* OSCC (42.1%; *p* = 0.07) [40]. Dhanda et al. showed that the combination of smooth muscle actin and SERPINE1 expression (4G/5G polymorphism) in CAF had superior sensitivity and specificity to MRI for the detection of ENE (sensitivity: 81%; specificity: 54%) [41]. Predictors from biopsies, such as DR-I/TILs-L, also had high sensitivity and accuracy (80% and 70%, *p* < 0.01), and adding cDOI > 10 mm (DR-I/TILs-L/cDOI > 10 mm) provided high sensitivity, specificity, and accuracy (70%, 55%, and 76%, respectively; *p* < 0.01); this could also be evaluated simply even in biopsies. In addition, histological features, such as non-TB-H/pDOI > 10 mm, in resections were revealed to be excellent negative predictors of ENE, with high specificity and accuracy. These two predictors could also predict all ENEs, including ENEmi.

This study has some limitations. First, the sample size was relatively small, particularly in patients with ENE. Second, we did not perform survival analysis, and the prognostic value derived with the available predictors was unclear, although the presence of ENE is an established prognostic factor in patients with OSCC.

## Conclusions

Our study showed that TB-H was an independent risk factor for ENE because the TME status in primary OSCC was significantly associated with that in ENE. This is the first study to demonstrate the association among DR, TB, TILs, and DOI between primary OSCC and ENE. Histological evaluation of these factors could be included in pathological reports to provide more appropriate treatment for patients with OSCC. Further studies with a large number of patients are needed to establish the significance of DR, TB, and TILs, especially in biopsies, in patients with OSCC and to establish a useful and versatile nomogram using histopathological factors in biopsies for evaluating the presence of ENE in patients with OSCC.

## Supplementary Information

**Additional file 1:** Clinicopathological status of 186 surgically resected specimens. **Supplementary Table 1**. The clinicopathological features were evaluated as follows; Sex (0: female; 1: male); cDOI 5 mm (0: ≤5 mm; 1: <5 mm); cDOI 10 mm (0: ≤10 mm; 1: DOI >10 mm); pDOI 5 mm (0: ≤5 mm; 1: <5 mm); pDOI 10 mm (0: ≤10 mm; 1: DOI >10 mm); Metastasis (0: absence; 1: presence); Number of lymph node metastases (0: n=0; 1: n=1; 2: n≥2); Pattern of invasion (1: pattern of 1 and 2; 2: pattern of 3 and 4); LyV (0: absence; 1: presence); pT (0: pT1, 2; 1: pT3, 4); pN (0: no LN metastasis; 1: pN1; 2: pN2 and 3); ENE (0: absence; 1: presence); DR (0: DR-M; 1: DR-I); TB (0: TB-L; 1: TB-H); TILs (0: TILs-L; 1: TILs-H); TB-H/pDOI>10 mm (0: no; 1: yes); DR-I/TILs-L (0: no; 1: yes); DR-I/TILs-L/pDOI >10 mm (0: no; 1: yes). MRI and CT were used in measuring the cDOI. Each unknown mass and artifact indicates that MRI could not detect a mass due to the small size, superficial location, or artifact. cDOI, clinical depth of invasion; pDOI, pathological depth of invasion; pT, pathological T; pN, pathological N; ENE, extranodal extension; LyV, lymphovascular invasion; DR, desmoplastic reaction; TB, tumor budding; TILs, tumor-infiltrating lymphocytes; TB-H, high tumor budding; DR-I, immature desmoplastic reaction; TILs-L, low-grade tumor-infiltrating lymphocytes; MRI, magnetic resonance imaging; CT, computed tomography.

**Additional file 2.** Detection of high-risk HPV infection status. The detection methods for patients at high risk of HPV infection are described. We evaluated the RNA in 186 patients with OSCC using the RNA scope 2.0 BROWN assay kit (Advanced Cell Diagnostics, Hayward, CA, USA). HPV, human papilloma virus; OSCC, oral squamous cell carcinoma

**Additional file3.** Histopathological features of RNA *in situ* hybridization HPV-HR18. Positive control: The endocervical high-grade squamous intraepithelial lesion shows punctate brown signals localized to the nuclei and/or cytoplasm (A). Hematoxylin and eosin (H&E) staining, ×400, (B). RNA* in situ *hybridization, ×400*. *There are no signals in the oral squamous cell carcinoma (C). H&E staining, ×200, (D). RNA* in situ *hybridization, ×400

**Additional file 4.** Clinicopathological status of 83 biopsy specimens. The clinicopathological features were evaluated as follows; Sex (0: female; 1: male); cDOI 5 mm (0: ≤5 mm; 1:<5 mm); cDOI 10 mm (0: ≤10 mm; 1: DOI>10 mm); Metastasis (0: absence; 1: presence); Number of lymph node metastasis (0: n=0; 1: n=1; 2: n ≥2); Pattern of invasion (1: pattern of 1 and 2; 2: pattern of 3 and 4); LyV (0: absence; 1: presence); pT (0: pT1, 2; 1: pT3, 4); pN (0: no LN metastasis; 1: pN1; 2: pN2, and 3); ENE (0: absence; 1: presence); DR (0: DR-M; 1: DR-I); TB (0: TB-L; 1: TB-H); TILs (0: TILs-L; 1: TILs-H); TB-H/cDOI >10 mm (0: no; 1: yes); DR-I/TILs-L (0: no; 1: yes); DR-I/TILs-L/cDOI >10 mm (0: no; 1: yes).cDOI, clinical depth of invasion; pT, pathological T; pN, pathological N; ENE, extranodal extension; DR, desmoplastic reaction; TB, tumor budding; TILs, tumor-infiltrating lymphocytes; TB-H, high tumor budding; DR-I, immature desmoplastic reaction; TILs-L, low-grade tumor-infiltrating lymphocytes.

**Additional file 5.** Bivariate logistic regression analyses of ENE using biopsy specimens. In the biopsies, bivariate logistic regression analyses revealed no significant relationships between the above-mentioned risk factors and presence of ENE (all* p*>0.05). CI, confidence interval; inf, infimum; DR-I, immature desmoplastic reaction; TB-H, high tumor budding; TILs-L, low-grade tumor-infiltrating lymphocytes; cDOI, clinical depth of invasion; ENE, extranodal extension.

**Additional file 6.** Association of combination risk factors with the diameters of the extent of ENE and tumor deposits in lymph nodes. The figure shows the association of risk factors with the diameters of tumor deposits in the lymph nodes and extent of ENE using surgically resected specimens. We investigated whether TB-H/pDOI >10 mm, DR-I/TILs-L, and DR-I/TILs-L/cDOI>10 mm could predict ENE status regardless of the tumor deposit size or extent of ENE. Mann–Whitney *U* test indicated no significant association in sizes between the cohort of TB-H/pDOI >10 mm and the others, between DR-I/TILs-L and the others, and DR-I/Tils-L/cDOI >10 mm and the others. Moreover, both DR-I/TILs-L and DR-I/TILs-L/cDOI >10 mm in the biopsy specimens could identify all ENEmi (100%, 5/5). *p-value: Comparison of semiquantitative values between the risk factor and other (non-risk factor) groups (Mann−Whitney *U* test). TB-H, high tumor budding; pDOI, pathological depth of invasion; DR-I, immature desmoplastic reaction; TILs-L, low-grade tumor-infiltrating lymphocytes; cDOI, clinical depth of invasion; ENE, extranodal extension; ENEmi, minor ENE.

## Data Availability

The datasets used and/or analyzed during the current study are available from the corresponding author on reasonable request.

## Abbreviations

AJCC: American Joint Committee on Cancer
CAF: Cancer-associated fibroblasts
cDOI: Clinical depth of invasion
cENE: Clinically defined extranodal extension
CI: Confidence interval
DFS: Disease-free survival
DOI: Depth of invasion
DR: Desmoplastic reaction
DR-I: Immature desmoplastic reaction
DR-M: Mature desmoplastic reaction
ENE: Extranodal extension
ENEmi: Minor ENE
H&E: Hematoxylin and eosin
HNSCC: Head and neck squamous cell carcinoma
HPV: Human papilloma virus
MRI: Magnetic resonance imaging
NPV: Negative predictive value
OR: Odds ratio
OSCC: Oral squamous cell carcinoma
pDOI: Pathological depth of invasion
PPV: Positive predictive value
pT: Pathological T
SCC: Squamous cell carcinoma
TB: Tumor budding
TB-H: High tumor budding
TB-L: Low tumor budding
TILs: Tumor-infiltrating lymphocytes
TILs-L: Low-grade tumor-infiltrating lymphocytes
TILs-H: High-grade tumor-infiltrating lymphocytes
TME: Tumor microenvironment

## References

[1] Bray F, Ferlay J, Soerjomataram I, Siegel RL, Torre LA, Jemal A (2018). Global cancer statistics 2018: GLOBOCAN estimates of incidence and mortality worldwide for 36 cancers in 185 countries. CA Cancer J Clin.

[2] ganjoho.jp Cancer statistics. https://ganjoho.jp/reg_stat/statistics/stat/cancer/3_oral.html Accessed 20 Jan 2022.

[3] Montero PH, Patel SG (2015). Cancer of the oral cavity. Surg Oncol Clin N Am.

[4] Shaw RJ, Lowe D, Woolgar JA, Brown JS, Vaughan ED, Evans C (2010). Extracapsular spread in oral squamous cell carcinoma. Head Neck.

[5] Lop J, Rigó A, Codina A, de Juan J, Quer M, León X (2018). Prognostic significance of extranodal extension in head and neck squamous cell carcinoma cN0 patients with occult metastatic neck nodes. Acta Otorrinolaringol Esp (Engl Ed).

[6] Ridge JA, Lydiatt WM, Patel SG, Amin MB, Edge S, Greene F (2017). Lip and oral cavity. AJCC cancer staging manual.

[7] Arun I, Maity N, Hameed S, Jain PV, Manikantan K, Sharan R (2021). Lymph node characteristics and their prognostic significance in oral squamous cell carcinoma. Head Neck.

[8] de Almeida JR, Truong T, Khan NM, Su JS, Irish J, Gilbert R (2020). Treatment implications of postoperative chemoradiotherapy for squamous cell carcinoma of the oral cavity with minor and major extranodal extension. Oral Oncol.

[9] Woolgar JA, Rogers SN, Lowe D, Brown JS, Vaughan ED (2003). Cervical lymph node metastasis in oral cancer: the importance of even microscopic extracapsular spread. Oral Oncol.

[10] Cooper JS, Pajak TF, Forastiere AA, Jacobs J, Campbell BH, Saxman SB (2004). Postoperative concurrent radiotherapy and chemotherapy for high-risk squamous-cell carcinoma of the head and neck. N Engl J Med.

[11] Bachaud JM, Cohen-Jonathan E, Alzieu C, David JM, Serrano E, Daly-Schveitzer N (1996). Combined postoperative radiotherapy and weekly cisplatin infusion for locally advanced head and neck carcinoma: final report of a randomized trial. Int J Radiat Oncol Biol Phys.

[12] Huang SH, O’Sullivan B (2017). Overview of the 8th edition TNM classification for head and neck cancer. Curr Treat Options Oncol.

[13] Chiu K, Hosni A, Huang SH, Tong L, Xu W, Lu L (2021). The potential impact and usability of the eighth edition TNM staging classification in oral cavity cancer. Clin Oncol (R Coll Radiol).

[14] Lydiatt WM, Patel SG, O’Sullivan B, Brandwein MS, Ridge JA, Migliacci JC (2017). Head and neck cancers-major changes in the American Joint Committee on cancer eighth edition cancer staging manual. CA Cancer J Clin.

[15] Tirelli G, de Groodt J, Sia E, Belgrano MG, Degrassi F, Boscolo-Rizzo P (2021). Accuracy of the Anatomage Table in detecting extranodal extension in head and neck cancer: a pilot study. J Med Imaging (Bellingham).

[16] Hiyama T, Kuno H, Nagaki T, Sekiya K, Oda S, Fujii S (2020). Extra-nodal extension in head and neck cancer: how radiologists can help staging and treatment planning. Jpn J Radiol.

[17] Pilborough AE, Lambert DW, Khurram SA (2019). Extranodal extension in oral cancer: a role for the nodal microenvironment?. J Oral Pathol Med.

[18] Quail DF, Joyce JA (2013). Microenvironmental regulation of tumor progression and metastasis. Nat Med.

[19] Heikkinen I, Bello IO, Wahab A, Hagström J, Haglund C, Coletta RD (2019). Assessment of tumor-infiltrating lymphocytes predicts the behavior of early-stage oral tongue cancer. Am J Surg Pathol.

[20] Badalamenti G, Fanale D, Incorvaia L, Barraco N, Listì A, Maragliano R (2019). Role of tumor-infiltrating lymphocytes in patients with solid tumors: can a drop dig a stone?. Cell Immunol.

[21] Lao XM, Liang YJ, Su YX, Zhang SE, Zhou XI, Liao GQ (2016). Distribution and significance of interstitial fibrosis and stroma-infiltrating B cells in tongue squamous cell carcinoma. Oncol Lett.

[22] Marsh D, Suchak K, Moutasim KA, Vallath S, Hopper C, Jerjes W (2011). Stromal features are predictive of disease mortality in oral cancer patients. J Pathol.

[23] Almangush A, Pirinen M, Heikkinen I, Mäkitie AA, Salo T, Leivo I (2018). Tumour budding in oral squamous cell carcinoma: a meta-analysis. Br J Cancer.

[24] Ueno H, Kanemitsu Y, Sekine S, Ishiguro M, Ito E, Hashiguchi Y (2017). Desmoplastic pattern at the tumor front defines poor-prognosis subtypes of colorectal cancer. Am J Surg Pathol.

[25] Lugli A, Kirsch R, Ajioka Y, Bosman F, Cathomas G, Dawson H (2017). Recommendations for reporting tumor budding in colorectal cancer based on the International Tumor Budding Consensus Conference (ITBCC) 2016. Mod Pathol.

[26] Hase K, Shatney C, Johnson D, Trollope M, Vierra M (1993). Prognostic value of tumor “budding” in patients with colorectal cancer. Dis Colon Rectum.

[27] Luo WR, Gao F, Li SY, Yao KT (2012). Tumour budding and the expression of cancer stem cell marker aldehyde dehydrogenase 1 in nasopharyngeal carcinoma. Histopathology.

[28] Teramoto H, Koike M, Tanaka C, Yamada S, Nakayama G, Fujii T (2013). Tumor budding as a useful prognostic marker in T1-stage squamous cell carcinoma of the esophagus. J Surg Oncol.

[29] Masuda R, Kijima H, Imamura N, Aruga N, Nakamura Y, Masuda D (2012). Tumor budding is a significant indicator of a poor prognosis in lung squamous cell carcinoma patients. Mol Med Rep.

[30] Liang F, Cao W, Wang Y, Li L, Zhang G, Wang Z (2013). The prognostic value of tumor budding in invasive breast cancer. Pathol Res Pract.

[31] Goel V, Parihar PS, Parihar A, Goel AK, Waghwani K, Gupta R (2016). Accuracy of MRI in prediction of tumour thickness and nodal stage in oral tongue and gingivobuccal cancer with clinical correlation and staging. J Clin Diagn Res.

[32] Ueno H, Kanemitsu Y, Sekine S, Ishiguro M, Ito E, Hashiguchi Y (2019). A multicenter study of the prognostic value of desmoplastic reaction categorization in Stage II colorectal cancer. Am J Surg Pathol.

[33] Ueno H, Murphy J, Jass JR, Mochizuki H, Talbot IC (2002). Tumour ‘budding’ as an index to estimate the potential of aggressiveness in rectal cancer. Histopathology.

[34] Suárez-Sánchez FJ, Lequerica-Fernández P, Rodrigo JP, Hermida-Prado F, Suárez-Canto J, Rodríguez-Santamarta T (2021). Tumor-infiltrating CD20^+^ B lymphocytes: significance and prognostic implications in oral cancer microenvironment. Cancers (Basel).

[35] Fu JY, Zhu L, Li J, Chen PQ, Shi WT, Shen SK (2021). Assessing the magnetic resonance imaging in determining the depth of invasion of tongue cancer. Oral Dis.

[36] El-Naggar AK, Chan JKC. WHO classification of head and neck tumours. 4th ed Grandis JR, Takata T, Grandis J, Slootweg PJ, editors. Lyon: IARC; 2017.

[37] Myers JN, Greenberg JS, Mo V, Roberts D (2001). Extracapsular spread. A significant predictor of treatment failure in patients with squamous cell carcinoma of the tongue. Cancer.

[38] Shibuya Y, Ohtsuki Y, Hirai C, Hasegawa T, Akashi M, Shigeta T (2014). Oral squamous cell carcinoma with microscopic extracapsular spread in the cervical lymph nodes. Int J Oral Maxillofac Surg.

[39] Huang SH, O’Sullivan B (2013). Oral cancer: current role of radiotherapy and chemotherapy. Med Oral Patol Oral Cir Bucal.

[40] Gleber-Netto FO, Neskey D, Costa AFM, Kataria P, Rao X, Wang J (2020). Functionally impactful TP53 mutations are associated with increased risk of extranodal extension in clinically advanced oral squamous cell carcinoma. Cancer.

[41] Dhanda J, Triantafyllou A, Liloglou T, Kalirai H, Lloyd B, Hanlon R (2014). SERPINE-1 and SMA expression at the invasive front predict extracapsular spread and survival in oral squamous cell carcinoma. Br J Cancer.

[42] Caldeira PC, Soto AML, de Aguiar MCF, Martins CC (2020). Tumor depth of invasion and prognosis of early-stage oral squamous cell carcinoma: a meta-analysis. Oral Dis.

[43] Huang SH, Hwang D, Lockwood G, Goldstein DP, O’Sullivan B (2009). Predictive value of tumor thickness for cervical lymph-node involvement in squamous cell carcinoma of the oral cavity: a meta-analysis of reported studies. Cancer.

[44] Zhang D, Song Y, Li D, Liu X, Pan Y, Ding L, Shi G, Wang Y, Ni Y, Hou Y. Cancer-associated fibroblasts promote tumor progression by lncRNA-mediated RUNX2/GDF10 signaling in oral squamous cell carcinoma. Mol Oncol. 2021. 2021;10.1002/1878-0261.12935. 10.1002/1878-0261.12935 PMC8807363 33657265

[45] Almangush A, Mäkitie AA, Triantafyllou A, de Bree R, Strojan P, Rinaldo A (2020). Staging and grading of oral squamous cell carcinoma: an update. Oral Oncol.

[46] de Abreu PM, Có ACG, Azevedo PL, do Valle IB, de Oliveira KG, Gouvea SA (2018). Frequency of HPV in oral cavity squamous cell carcinoma. BMC Cancer.

[47] Troiano G, Rubini C, Togni L, Caponio VCA, Zhurakivska K, Santarelli A, Cirillo N, Lo Muzio L, Mascitti M (2020). The immune phenotype of tongue squamous cell carcinoma predicts early relapse and poor prognosis. Cancer Med.

[48] Mascitti M, Zhurakivska K, Togni L, Caponio VCA, Almangush A, Balercia P, Balercia A, Rubini C, Lo Muzio L, Santarelli A, Troiano G (2020). Addition of the tumour-stroma ratio 8th edition to the American Joint Committee on Cancer staging system improves survival prediction for patients with oral tongue squamous cell carcinoma. Histopathology.

[49] Almangush A, Bello IO, Heikkinen I, Hagström J, Haglund C, Kowalski LP, Nieminen P, Coletta RD, Mäkitie AA, Salo T, Leivo I (2021). Stromal categorization in early oral tongue cancer. Virchows Arch.

