# Supplementary material for: Novel pathological predictive factors for extranodal extension in oral squamous cell carcinoma: a retrospective cohort study based on tumor budding, desmoplastic reaction, tumor-infiltrating lymphocytes, and depth of invasion
Source: BMC Cancer. 2022 Apr 13;22:402. doi: 10.1186/s12885-022-09393-8 (PMC9006434; doi:10.1186/s12885-022-09393-8)
Supplement: Supplementary file 2 — Additional file 2. Detection of high-risk HPV infection status. The detection methods for patients at high risk of HPV infection are described. We evaluated the RNA in 186 patients with OSCC using the RNA scope 2.0 BROWN assay kit (Advanced Cell Diagnostics, Hayward, CA, USA). HPV, human papilloma virus; OSCC, oral squamous cell carcinoma [file 12885_2022_9393_MOESM2_ESM.doc]

**Additional File 2**

Human papillomavirus (HPV) infection has been identified in ~5% of oral squamous cell carcinoma (OSCC) cases [1] and implicated in extranodal extension development and tumor behavior. Therefore, we determined the HPV status of the participants to exclude the bias underlying molecular etiology. Tissue microarrays (TMAs) were obtained from 186 patients with OSCC from the paraffin-embedded resected tumor block and punched out with a 2-mm biopsy needle. *In situ* hybridization was performed for all 186 patients with OSCC using the TMA specimens; they were evaluated using the RNA scope 2.0 BROWN assay kit (Advanced Cell Diagnostics, Hayward, CA, USA) and HPV-HR18 probe cocktail (Advanced Cell Diagnostics, Hayward, CA, USA) in accordance with the manufacturer’s instruction. Staining data were recorded according to the thickness of epithelial staining, presence and extent of diffuse and/or punctate nuclear staining and cytoplasmic staining, and signal intensity. The controls in each staining run included a known HPV-positive lesion, high-grade squamous intraepithelial lesion, SCC of the uterine cervix, SCC of the oropharynx as a positive control, and a section of normal tonsil as a negative control. Positive staining was identified as any of the tumor cells showing brown punctate dot-like nuclear and/or cytoplasmatic positivity (Additional File 3). Cases were classified in a binary manner as either positive or negative [2].

**References**

**1.** de Abreu PM, Có ACG, Azevedo PL, do Valle IB, de Oliveira KG, Gouvea SA, et al. Frequency of HPV in oral cavity squamous cell carcinoma. BMC Cancer. 2018;18:324.

**2.** Mirghani H, Casiraghi O, Amen F, He M, Ma XJ, Saulnier P, et al. Diagnosis of HPV-driven head and neck cancer with a single test in routine clinical practice. Mod Pathol. 2015;28:1518-27.
