# Supplementary figures and images for: Novel pathological predictive factors for extranodal extension in oral squamous cell carcinoma: a retrospective cohort study based on tumor budding, desmoplastic reaction, tumor-infiltrating lymphocytes, and depth of invasion
Source: BMC Cancer. 2022 Apr 13;22:402. doi: 10.1186/s12885-022-09393-8 (PMC9006434; doi:10.1186/s12885-022-09393-8)

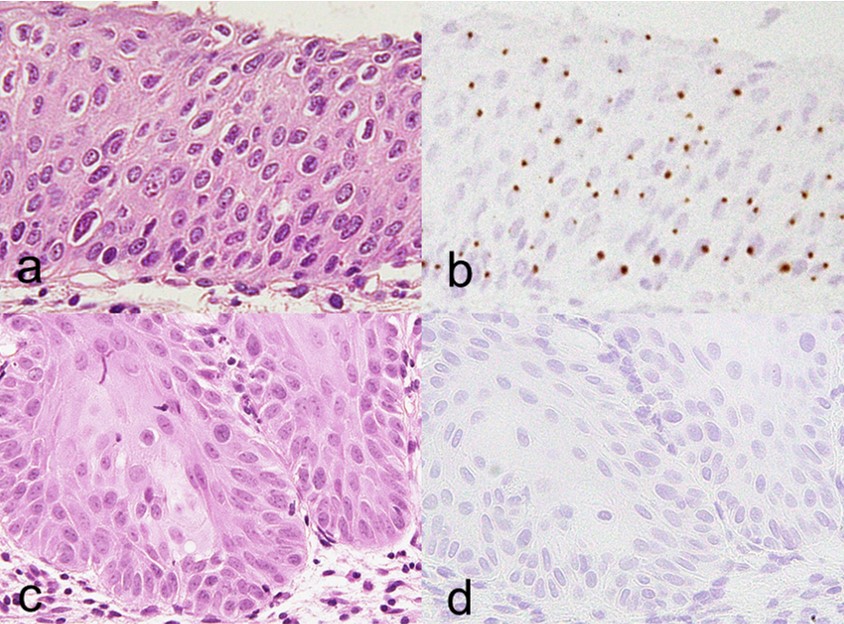

Supplement: Supplementary file 3 — Additional file3. Histopathological features of RNA in situ hybridization HPV-HR18. Positive control: The endocervical high-grade squamous intraepithelial lesion shows punctate brown signals localized to the nuclei and/or cytoplasm (A). Hematoxylin and eosin (H&E) staining, ×400, (B). RNA in situ hybridization, ×400. There are no signals in the oral squamous cell carcinoma (C). H&E staining, ×200, (D). RNA in situ hybridization, ×400 [file 12885_2022_9393_MOESM3_ESM.jpg]

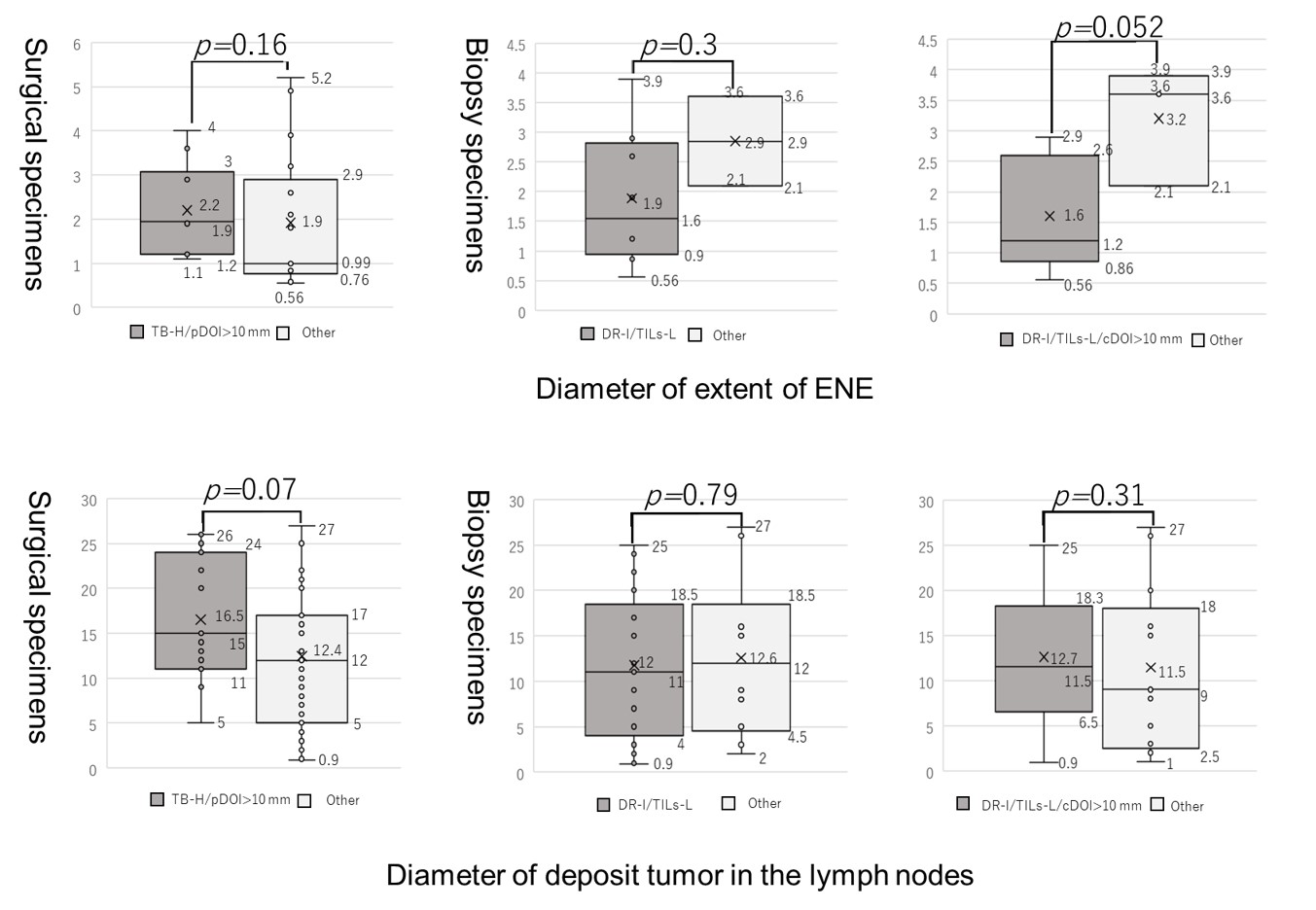

Supplement: Supplementary file 6 — Additional file 6. Association of combination risk factors with the diameters of the extent of ENE and tumor deposits in lymph nodes. The figure shows the association of risk factors with the diameters of tumor deposits in the lymph nodes and extent of ENE using surgically resected specimens. We investigated whether TB-H/pDOI >10 mm, DR-I/TILs-L, and DR-I/TILs-L/cDOI>10 mm could predict ENE status regardless of the tumor deposit size or extent of ENE. Mann–Whitney U test indicated no significant association in sizes between the cohort of TB-H/pDOI >10 mm and the others, between DR-I/TILs-L and the others, and DR-I/Tils-L/cDOI >10 mm and the others. Moreover, both DR-I/TILs-L and DR-I/TILs-L/cDOI >10 mm in the biopsy specimens could identify all ENEmi (100%, 5/5). *p-value: Comparison of semiquantitative values between the risk factor and other (non-risk factor) groups (Mann−Whitney U test). TB-H, high tumor budding; pDOI, pathological depth of invasion; DR-I, immature desmoplastic reaction; TILs-L, low-grade tumor-infiltrating lymphocytes; cDOI, clinical depth of invasion; ENE, extranodal extension; ENEmi, minor ENE. [file 12885_2022_9393_MOESM6_ESM.jpg]
