# Supplementary material for: Novel pathological predictive factors for extranodal extension in oral squamous cell carcinoma: a retrospective cohort study based on tumor budding, desmoplastic reaction, tumor-infiltrating lymphocytes, and depth of invasion
Source: BMC Cancer. 2022 Apr 13;22:402. doi: 10.1186/s12885-022-09393-8 (PMC9006434; doi:10.1186/s12885-022-09393-8)
Supplement: Supplementary file 5 — Additional file 5. Bivariate logistic regression analyses of ENE using biopsy specimens. In the biopsies, bivariate logistic regression analyses revealed no significant relationships between the above-mentioned risk factors and presence of ENE (all p>0.05). CI, confidence interval; inf, infimum; DR-I, immature desmoplastic reaction; TB-H, high tumor budding; TILs-L, low-grade tumor-infiltrating lymphocytes; cDOI, clinical depth of invasion; ENE, extranodal extension. [file 12885_2022_9393_MOESM5_ESM.doc]

**Additional File 5: Bivariate logistic regression analyses of ENE using biopsy specimens**

|  | Bivariate | | |
| --- | --- | --- | --- |
|  | Odds ratio | 95% CI | *p-value* |
| All (n=83) |  |  |  |
| DR-I | 2.15e+08 | 0.16-inf | 0.99 |
| TB-H | 7.24e-01 | 0.16-3.32 | 0.68 |
| TILs-L | 4.64e-01 | 0.08-2.81 | 0.40 |
| cDOI >5 mm | 5.76e-09 | 0.0-inf | 0.99 |
| cDOI >10 mm | 2.41e-09 | 0.00-inf | 0.99 |
| Progressive (n=47) |  |  |  |
| DR-I | 1.71e+08 | 0.00-inf | 0.99 |
| TB-H | 8.30e-01 | 0.17-4.06 | 0.81 |
| TILs-L | 9.10e-01 | 0.13-6.34 | 0.92 |
| cDOI >5 mm | 6.40e-09 | 0.00-inf | 0.99 |
| cDOI >10 mm | 1.21e+08 | 0.00-inf | 0.99 |
| Metastasis (n=27) |  |  |  |
| DR-I | 1.51e+08 | 0.00-inf | 0.99 |
| TB-H | 4.20e-01 | 0.06-3.01 | 0.39 |
| TILs-L | 5.06e-01 | 0.06-4.61 | 0.54 |
| cDOI >5 mm | 5.70e-09 | 0.00-inf | 0.99 |
| cDOI >10 mm | 2.37e+08 | 0.00-inf | 0.99 |

CI, confidence interval; inf, infimum; DR-I, immature desmoplastic reaction; TB, tumor budding; TILs-L, low-grade tumor-infiltrating lymphocytes; cDOI, clinical depth of invasion; LN, lymph node; ENE, extranodal extension.
